# Supplementary material for: Nanopore Sequencing for T-Cell Receptor Rearrangement Analysis in Cutaneous T-Cell Lymphoma
Source: Cancers (Basel). 2024 Nov 1;16(21):3700. doi: 10.3390/cancers16213700 (PMC11544856; doi:10.3390/cancers16213700)
Supplement: Supplementary file 1 [file cancers-16-03700-s001.zip › cancers-3235044-supplementary.pdf]

Supplements:

Table S1: Patient information from FFPE samples from CTCL patients (n=22) and other polyclonal manifestations (n=5).

Sample ID	Sex	Age	TNMB	Stage	Clinical manifestation	Diagnosis	TRG ONT TCF	TRG MiSeq TCF	TRB ONT TCF	TRB MiSeq TCF
1	M	77	$T_3N_0M_0B_0$	IIB	Tumor	Mycosis fungoides	0,77	0,79	N/A*	N/A*
2	M	55	$T_3N_3M_0B_0$	IVA2	Tumor	Mycosis fungoides	0,66	0,70	0,91	0,92
3	M	56	$pT_4N_2M_0B_2$	IVA1	Erythroderma	Sezary syndrome	0,63	0,63	0,56	0,69
4	M	76	$pT_3N_0M_0B_0$	IIB	Tumor	Mycosis fungoides	0,59	0,67	0,67	0,70
5	M	83	$T_3N_0M_0B_0$	IIB	Tumor	Mycosis fungoides	0,56	0,63	0,66	0,70
6	M	59	$T_3N_0B_0M_0$	IIB	Tumor	Epidermotrophic CTCL	0,55	0,55	0,11	0,10
7	F	86	$T_4N_0M_0B_2$	IVA1	Erythroderma	Sezary syndrome	0,45	0,48	0,22	0,24
8	M	80	$T_3N_0B_0M_0$	IIB	Tumor	Mycosis fungoides	0,37	0,34	N/A*	N/A*
9	M	56	$T_{1b}N_0M_0B_0$	IA	Plaque	Mycosis fungoides	0,89	0,94	0,46	0,49
10	M	84	$T_{1b}N_xM_xB_x$	IB	Plaque	Mycosis fungoides	0,79	0,82	N/A*	N/A*
11	M	46	$T_{1b}N_0B_0M_0$	IA	Plaque	Mycosis fungoides	0,53	0,60	0,66	0,72
12	F	66	$T_{1b}N_0M_0B_1$	IB	Plaque	Mycosis fungoides	0,50	0,48	N/A*	N/A*
13	F	51	$T_{1a}N_0M_0B_0$	IA	Patch	Mycosis fungoides	0,48	0,57	0,65	0,64
14	M	59	$T_{1a}B_0M_0N_0$	IA	Patch	Mycosis fungoides	0,39	0,46	0,31	0,35
15	M	49	$T_{2b}N_0M_0B_1$	IB	Plaque	Mycosis fungoides	0,35	0,39	0,14	0,14
16	F	75	$T_{1b}N_0M_0B_1$	IA	Plaque	Mycosis fungoides	0,32	0,31	0,38	0,45
17	M	57	$T_{1b}N_0M_0B_0$	IA	Plaque	Mycosis fungoides	0,28	0,35	0,03	0,03
18	M	66	$T_{1a}N_0M_0B_0$	IA	Patch	Mycosis fungoides	0,27	0,33	0,06	0,07
19	M	71	$T_{1b}N_0M_0B_0$	IB	Plaque	Mycosis fungoides	0,20	0,22	0,29	0,40
20	M	68	$T_{1a}N_0M_0B_0$	IA	Patch	Mycosis fungoides	0,18	0,20	0,05	0,05
21	M	80	$T_{1b}N_0M_0B_0$	IA	Plaque	Mycosis fungoides	0,05	0,06	0,07	0,09
22	M	61	$T_{1a}N_xM_xB_x$	IA	Patch	Mycosis fungoides	0,02	0,02	0,07	0,07
23	M	72	-	-	-	Pityriasis lichenoides chronica	0,05	0,06	0,02	0,02
24	F	50	-	-	-	Follicular B-cell lymphoma	0,04	0,05	0,03	0,03
25	F	73	-	-	-	Chronic dermatitis	0,03	0,03	0,03	0,04
26	F	26	-	-	-	Chronic dermatitis	0,02	0,02	0,05	0,05
27	M	71	-	-	-	Epidermotrophic drug exanthema	0,01	0,02	0,06	0,05

*N/A. = not available

Table S2: Patient information of 9 fresh and CD3-cell isolated samples CD3_1 – CD3_9) and 9 Fresh Frozen samples (FF_1 – FF_9) of MF patients.

Sample ID	Sex	Age	TNMB	Stage	Clinical manifes-tation	Diagnosis	TRG ONT TCF	TRG MiSeq TCF	TRB ONT TCF	TRB MiSeq TCF
CD3_1	M	74	$T_{1b}N_0M_0B_0$	IA	Patch	Mycosis fungoides	2,28	2,45	2,41	2,92
CD3_2	F	63	$T_{1a}N_0M_0B_0$	IA	Patch	Mycosis fungoides	18,90	14,77	3,83	4,54
CD3_3	M	74	$T_3N_0M_0B_0$	IIB	Tumor	Mycosis fungoides	55,40	61,61	13,92	19,09
CD3_4	M	72	$T_{2b}N_0M_0B_1$	IA	Patch	Mycosis fungoides	1,02	1,01	1,33	1,24
CD3_5	M	61	$T_2N_0M_0B_1$	IB	Plaque	Mycosis fungoides	2,13	2,07	1,46	1,53
CD3_6	M	64	$T_{1a}N_0M_0B_0$	IA	Patch	Mycosis fungoides	5,05	5,60	10,03	11,06
CD3_7	M	63	$T_{1a}N_0M_0B_0$	IA	Patch	Mycosis fungoides	45,40	53,72	2,00	1,85
CD3_8	M	60	$T_{1a}N_0M_0B_0$	IA	Patch	Mycosis fungoides	21,88	18,67	39,44	41,82
CD3_9	M	68	$T_{1a}N_0M_0B_0$	IA	Patch	Mycosis fungoides	41,46	46,42	9,04	10,65
FF_1	F	51	$T_3N_0M_0B_0$	IIB	Tumor	Mycosis fungoides	16,11	15,16	1,85	1,77
FF_2	M	84	$T_1N_0M_0B_1$	IB	Plaque	Mycosis fungoides	63,81	62,03	65,32	79,54
FF_3	F	52	$T_3N_0M_0B_0$	IIB	Tumor	Mycosis fungoides	48,12	42,50	11,83	10,92
FF_4	M	71	$T_3N_xM_0B_0$	IIB	Tumor	Mycosis fungoides	57,77	58,09	39,10	46,95
FF_5	F	88	N/A.*	IIB	Tumor	Mycosis fungoides	73,18	75,50	64,81	65,37
FF_6	F	56	$T_3N_0M_0B_0$	IIB	Tumor	Mycosis fungoides	35,91	33,18	11,63	11,54
FF_7	F	55	$T_3N_1M_0B_1$	IIB	Tumor	Mycosis fungoides	96,96	99,33	80,19	99,10
FF_8	M	57	$T_3N_xM_0B_0$	IIB	Tumor	Mycosis fungoides	83,86	87,66	7,23	6,43
FF_9	M	53	$T_{2b}N_0M_0B_0$	IB	Plaque	Mycosis fungoides	38,19	39,28	22,75	20,56

*N.A. = not available
